# Supplementary material for: The Role of Homogeneous Waiting Group Criteria in Patient Referrals: Views of General Practitioners and Specialists in South Tyrol, Italy
Source: Healthcare (Basel). 2024 May 10;12(10):985. doi: 10.3390/healthcare12100985 (PMC11120922; doi:10.3390/healthcare12100985)
Supplement: Supplementary file 1 [file healthcare-12-00985-s001.zip › healthcare-2921989-supplementary.pdf]

File S1. Homogeneous waiting group criteria for first gynecological consultation in Italy.

Priority Class	Maximum Waiting Time	Clinical Indications Recommended by the Working Group
Emergency	Send to emergency room	Acute pelvic pain<br>Hypertension in pregnancy<br>Severe menometrorrhagia<br>Blood loss in pregnancy
Emergency (pediatrics)	Send to emergency room	Acute pelvic pain<br>Severe menometrorrhagia<br>Suspected sexual abuse
Urgent – U	3 days	Ascites from probable gynecological pathology<br>Bartholinitis<br>Persistent vaginitis resistant to therapy<br>Other (10%)**
Urgent – U (pediatrics)	3 days	Ascites from probable gynecological pathology<br>Bartholinitis<br>Pregnancy in a minor<br>Persistent vaginitis resistant to therapy<br>Other (10%)*
Short – B	10 days	Breast lump<br>"Positive" Pap Test<br>Atypical blood losses (excluding severe menometrorrhagia)<br>Signs/symptoms of probable sexually transmitted disease<br>Suspected gynecological neoplasm<br>Other (10%)*
Short – B (Pediatrics)	10 days	Atypical blood losses (excluding severe menometrorrhagia)<br>Genital bleeding in pre-pubescence<br>Suspected gynecological neoplasm<br>Other (10%)*
Deferrable – D	30 days	Amenorrhea with negative pregnancy test<br>Ovarian cyst<br>Chronic pelvic pain<br>Uterine fibroids<br>Urinary incontinence<br>Utero-vaginal prolapse<br>Suspected genital condylomatosis<br>Assessment for contraception Other (10%)*
Deferrable – D (Pediatrics)	30 days	Amenorrhea with negative pregnancy test<br>Chronic pelvic pain<br>Other (10%)*
Programmable – P	120 days	Acne/Hirsutism<br>Symptomatic menopause<br>Couple infertility/infertility

		Other (10%)*
Programmable – P (Pediatrics)	120 days	Acne/Hirsutism<br>Other (10%)*

First gynecologic visit Class HWG 036 – Code 89.26.1 including any cytologic sampling, any indications in contraceptive or pre-conceptive function. Cannot be associated first obstetrical visit – Code 89.26.3 including eventual cytologic sampling which cannot be associated with first gynecological visit, respectively. *Any clinical condition not included in the recommended clinical indications that is considered to warrant assignment to that specific grouping; it presupposes in each case the detailed description of the clinical conditions.

Table S1. Summary of questionnaire items for general practitioners on collaboration, communication, and referral practices.

Item No.	Category	Description	Response Option
1	Collaboration and Communication	General satisfaction with collaboration and communication with hospital physicians.	a) Very satisfied<br>b) Satisfied<br>c) Dissatisfied<br>d) Very dissatisfied
2	Collaboration and Communication	Frequency of phone contact with hospital colleagues for clarifications.	a) Often<br>b) Occasionally<br>c) Rarely<br>d) Never
3	Collaboration and Communication	Frequency of contacting hospital colleagues via email about patients.	a) Often<br>b) Occasionally<br>c) Rarely<br>d) Never
4	Referral Practices	Frequency of avoiding or expediting specialist referrals through phone contact.	a) Often<br>b) Occasionally<br>c) Rarely<br>d) Never
5	Referral Practices	Estimated percentage of acute consultation reasons requiring specialist referral.	a) 80%<br>b) 50%<br>c) 30%<br>d) 10%
6	Influencing Factors for Referral	Factors influencing decision to refer patients to specialists. (Multiple responses allowed)	a) Severity of symptoms<br>b) Urgency of treatment<br>c) Unclear diagnosis<br>d) Patient request<br>e) Limited consultation time<br>f) Other (specify)
7	Impact of HWG Criteria	Impact of HWG[1] criteria on waiting times.	a) Facilitated<br>b) Made more difficult<br>c) Reduced waiting times<br>d) Extended waiting times<br>e) Unchanged waiting times
8	Impact of HWG Criteria	Frequency of prescribing priority visits/exams.	a) Less than 5%<br>b) 10%

			c) 20%<br>d) 30%<br>e) Other (specify)
9	Impact of HWG Criteria	Agreement with RAO criteria and accessibility.	a) Completely agree<br>b) Mostly agree<br>c) Partly agree<br>d) Do not agree at all
10	Impact of HWG Criteria	Adherence to RAO criteria in prescriptions.	a) 90%<br>b) 70%<br>c) 50%<br>d) Less than 30%
11	Non-compliance with HWG Criteria	Common reasons for non-compliance with RAO criteria.	a) Long waits for normal appointments<br>b) Clinical urgency not matching RAO<br>c) Conditions unresponsive to conventional therapy<br>d) Unclear clinical picture not fitting RAO<br>e) Patient or family pressure<br>f) Other (specify)
12	Non-compliance with HWG Criteria	Disciplines/areas needing changes in priority criteria.	Open-ended response
13	Adherence to Expected Waiting Times and Specialties with Adherence Issues	Adherence to expected waiting times based on priority criteria.	a) Absolutely<br>b) Mostly<br>c) No, not really<br>d) Not at all
14	Adherence to Expected Waiting Times and Specialties with Adherence Issues	Specialties with particular adherence issues to waiting times.	Open-ended response
15	Opinions and Actions Related to Non-Urgent Appointments and Delayed Priority Specialist Visits	Opinion on waiting times for non-urgent appointments.	a) Completely long<br>b) Mostly long<br>c) Not really long<br>d) Not long at all
16	Opinions and Actions Related to Non-Urgent Appointments and Delayed Priority Specialist Visits	Actions taken when a patient requires a delayed priority specialist visit.	a) Suggest private specialist/facility<br>b) Arrange earlier hospital appointment<br>c) Prescribe urgent visit and direct to ER<br>d) Advise direct ER visit without referral
…[2]	Other	Various questions/topic	Various response options

[1]HWG, Homogeneous Waiting Grouping that allows for different timings for access to outpatient specialist services based on explicit clinical indications. [2]Detailed descriptions and response options for additional questions classified under "Other" are available upon request.

Table S2. Summary of questionnaire items for hospital physicians on collaboration, communication, and referral practices.

Item No.[1]	Category	Description	Response Options
1	Collaboration and Communication	General satisfaction with collaboration and communication with GPs.	a) Very satisfied<br>b) Satisfied<br>c) Dissatisfied<br>d) Very dissatisfied
2	Collaboration and Communication	Ability to contact GPs by phone for any questions.	a) Often<br>b) Occasionally<br>c) Rarely<br>d) Never
3	Collaboration and Communication	Suggestions for reducing waiting times.	Open-ended response
4	Collaboration and Communication	Extent of connectivity between your hospital department and general medicine.	a) Very well connected<br>b) Well connected<br>c) Poorly connected<br>d) Very poorly connected
5	Collaboration and Communication	Importance of establishing a fixed phone number and/or email for each department, accessible to GPs.	a) Very important<br>b) Important<br>c) Not very important<br>d) Not important at all
6	Collaboration and Communication	Additional comments or suggestions for improving collaboration and communication with GPs and the specialist referral system.	Open-ended response
7	Collaboration and Communication	Evaluation of the current work climate and collaboration with colleagues from other disciplines in the hospital.	a) Very good<br>b) Good<br>c) Fair<br>d) Poor<br>e) Very poor
8	Collaboration and Communication	Additional comments or suggestions for improving collaboration with colleagues from other hospital disciplines.	Open-ended response
9	Referral Practices	Frequency of seeing patients in priority or urgent visits sent by a GP, which, in your opinion, could have been normal, non-urgent visits.	a) Less than 10%<br>b) 10-30%<br>c) 30-60%<br>d) More than 60%
10	Referral Practices	Frequency of providing feedback to GPs regarding inappropriate referrals.	a) Very often<br>b) Often<br>c) Occasionally<br>d) Rarely<br>e) Never
11	Referral Practices	Necessity of creating open slots in specialist clinics for patients with particular problems, accessible through direct phone contact by GPs.	a) Very necessary<br>b) Necessary<br>c) Unnecessary<br>d) Very unnecessary
12	Referral Practices	Evaluation of the indication of clinical questions on the referrals	a) Very good<br>b) Good

		from General Practitioners for specialist visits.	c) Fair<br>d) Poor<br>e) Very poor
13	Referral Practices	Frequency of patients receiving an inappropriate referral for a specialist visit due to patient requests.	a) Very often<br>b) Often<br>c) Occasionally<br>e) Never
14	Referral Practices	Common reasons for inappropriate referrals to hospital specialists.	Open-ended response
15	Referral Practices	Factors frequently influencing GPs' decisions to refer patients to specialists and the urgency level. (Multiple responses allowed)	a) Severity of symptoms<br>b) Urgency of treatment<br>c) Unclear diagnosis<br>d) Patient request<br>e) Limited consultation time<br>f) Other (please explain)
16	Impact of HWG[1] Criteria	Level of awareness about current guidelines and HWG criteria for priority patient referrals by GPs.	a) Very well informed<br>b) Well informed<br>c) Fairly informed<br>d) Poorly informed
17	Adherence to Expected Waiting Times and Specialties with Adherence Issues	Opinion on whether waiting times for normal, deferrable visits are generally too long.	a) Yes, absolutely<br>b) Yes, mostly<br>c) No, not really<br>d) No, not at all
18	Opinions and Actions Related to Non-Urgent Appointments and Delayed Priority Specialist Visits	Belief that improving HWG priority criteria by GPs could reduce the number of inappropriate referrals.	a) Yes, certainly<br>b) Yes, probably<br>c) No, probably not<br>d) No, not at all
19	Opinions and Actions Related to Non-Urgent Appointments and Delayed Priority Specialist Visits	Overall satisfaction with the current patient referral system to specialists.	a) Very satisfied<br>b) Satisfied<br>c) Dissatisfied<br>d) Very dissatisfied
20	Opinions and Actions Related to Non-Urgent Appointments and Delayed Priority Specialist Visits	Favorability towards the development of alternative care methods, such as telemedicine.	a) Yes, definitely<br>b) Yes, probably<br>c) No, probably not<br>d) No, not at all

[1]HWG, Homogeneous Waiting Groupings that allows for different timings for access to outpatient specialist services based on explicit clinical indications.

Table S3. General practitioner perspectives on proposals for improvement of communication and collaboration.

Item	Response	Rate (%)[1]
How important is it to you that health agency communications include a brief summary of the organizational changes?	Very important or important	80.5
	Less important or not important	12.2
How important would it be to you for the health agency to create a link where you can find all the forms, circulars and regulations that affect you as a GP?	Very important or important	89.1
	Less important or not important	3.6
How important would it be for you to have a telephone number for initial consultations and examinations and a number for follow-up visits for GPs only?	Very important or important	70.8
	Less important or not important	22.0
How important would it be to you that the Health Agency's primary care services are led by experienced general practitioners rather than specialists in hygiene?	Very important Important	86.6
	Less important Not important	5.1
Should 3 to 4 annual meetings be organized with the medical management of the primary care services at district level in order to address problems in good time and discuss possible solutions?	Yes, definitely or yes, but less often	83.0
	No, rather not or no, not at all	7.3
How important is it to you that every specialist in the hospital has a fixed telephone number where they can be reached daily and where they can guarantee to call you back if they cannot be reached?	Very important or important	72.0
	Less important or not important	19.5

[1] No indication of missing answers.
